# Supplementary material for: Patterns of Intron Gain and Loss in Fungi
Source: PLoS Biol. 2004 Nov 30;2(12):e422. doi: 10.1371/journal.pbio.0020422 (PMC532390; doi:10.1371/journal.pbio.0020422)
Supplement: Table S1 — Also available at http://genes.mit.edu/NielsenEtAl/. (4.3 MB ZIP). [file pbio.0020422.st001.zip › NielsenEtAl/html/707.html]

AN0093.1.NCU06271.1.MG05259.1.FG02562.1


```
 CLUSTAL W (1.82) Multiple Sequence Alignments - Introns Inserted


Sequence 1: MG05259.1	857 aa
Sequence 2: FG02562.1	859 aa
Sequence 3: NCU06271.1	896 aa
Sequence 4: AN0093.1	798 aa
Alignment Length: 935 aa
Number Identitical Residues: 184 aa
Alignment Score (without introns) 10725


MG05259.1 	MSPRKRARRDPTEANTSRPLIPSTSSSK---PPTALPTRAVSSSHAS~ASSPALPTTFAP
NCU06271.1	MGRRKKAKPNPPTEPADGSAVTESSTSTSDMPPKSSSSLAASIWRSK~AVP--MPTASNP
FG02562.1 	-----MTKDDANTLVSTSTIQTPTSQSQ---APKASPATESEQGNSQ~KVPSRKQTMSDS
AN0093.1  	-----------MASPASRKRVKPSPQSP---DVTSPNSKGSNSGRSS0WYPSVWPSKAAP
          	               :       :. *      .:  :   .  .:.   ..   :   .

MG05259.1 	FSAATAASASASRLSLVHDAQQQQQQPPVKQVHKS~R~SWYGSWPRAP~RSKAGASTQVA
NCU06271.1	PSSTTTSTPDN-ALSTSQPNATANTTPISSKRGLK~E0TRSPRNLGVE1R--------ST
FG02562.1 	CSTERRISKDVGVLGLYLTLLRSNKTCRYKDQGVV1K~SKKS--PDLS~RYEAKR-NDSD
AN0093.1  	ITEVARESISMGKHTPS-DAASESTIPLYSPKQ--~-~----------~----------Q
          	 :     : . .           .     .                              

MG05259.1 	RESILGGTLKPDTTPNLTRFDTKRSTDSDSDTPGSLSQLPPVEELDTPTGHRHPS-----
NCU06271.1	DSVSLDGAASSSSLPNMLPSE---NLDDRGSTGGEVPKPGPAVDDNVAKTDQVAVSTTGS
FG02562.1 	DASILSTTGTIKTMPNIKQNKSDVTMTEEVAQKESASQIKPDVELSREPAQITESQETSK
AN0093.1  	RNPSLQLTKRAGGLTRSLPAS---ATTTRINIASDGSTSGPAEEQPDPTSSGKTA-----
          	    *  :      ..    .             . .   *  :                

MG05259.1 	DSGAAEPNGHTNTKSDDATSATAAGEDDIPPLPELQRPTGVAELPRRPATSSGWLGWLGR
NCU06271.1	NEAEDRPSPDTQQQQQQQQPTSDTNGIHQPADSQSTTTEP----QSGETASSSWLGWLGQ
FG02562.1 	DAGTAETTTHETQESETSDAQSKSQADMRPAASASQNDD------QQPPTSSGWLGWWSR
AN0093.1  	DTDDKEISKDTSAIKAANSEDHQSDPTGEPAVSSNLDAA--------VTQPAGWLSWVYA
          	:    . . .    .        :     *. .               . .:.**.*   

MG05259.1 	SAAP-APVQTVESTSIPENAKAQNPEAKPLLAPDS--SKDDSK--SQNGNPGYDTISTSA
NCU06271.1	SAYYYYPVLTASVPVLPKLSEPRPNGGEPEVPEPPKLTEESSNNMPSDGDPPKAPDAQEP
FG02562.1 	-----TPFTETQATPTVDTS------TQPMIEIEA--TKEADT--PRPITP---PAQVEP
AN0093.1  	PFVYNKTTASYSTAETTTEQT-----AEPEIPAADHSSNQDNESAPSEPSPEQDKPSEVT
          	.     .    . .             :* :      ::: . . .    *        .

MG05259.1 	SLHPSSSESPAEPAATSWFGFWPTSRTAPPQEATSVKSDIEQNTKPLSSDPVTEPIKNRE
NCU06271.1	TLQTAPQEKPTYSYGSYLFGFWPGSGTTETKQTDSALSTGEPQ-----EGATSPPSKDSE
FG02562.1 	SEQMTSPKNEQDPRPTSWFRYWYPS--SEPAKAPEDKPNIQQ--------PQIETEPPPE
AN0093.1  	EITSTSQKR---SWLQMWYGSSPTKGPKAFKDEASCNAQEQHP------PAPVASSRSDE
          	    :. :    .     :     . .    .  .  .  :         .   .    *

MG05259.1 	DVVMSDN---SVTSPEAQP---AGSTWAFWSRD-PNTKSTGSGKGVAKREPGEIAVIGEG
NCU06271.1	DVEMEDAPAVEAAKPAPAQQPKSGSTWAFWYRDSGSNSHTKDGQ---KEETGQLAVVGES
FG02562.1 	DVVMKDA---EPPSRQNEPPPKSGSTWAFWSKESPKTKESQP-----LPESGEVAVMGEG
AN0093.1  	DTERTRTEG-GTDTPKSTNTSTKPSAWSFFFRDSAQERTQEA-------QATETTIIQD-
          	*.      .    .      ..  *:*:*: ::: .             :. : ::: : 

MG05259.1 	SEAHP------EPASTVDNAPP-AAPSAVSKTKELPK--------KSKRVRAQSMDIDYP
NCU06271.1	SESQPKNANVTEPETEPSKEPSLKSVKKDDQPRAATPSLKESSSNKGKRARPQSM-VDEP
FG02562.1 	SEARP------QPMAEFDVSPTKYSKIEDSKAKDKPASVKSRWMTKNKRVRPQSMDLDHT
AN0093.1  	------------PLAEQS------TSVSDSKAG-----------RKTEVINKGNVKVNAP
          	            * :  .      :    .:.             * :  .  .:.:: .

MG05259.1 	TT--ANRPSTATSVATTKTESSIKAATPAVDG-------AAKSSAGNLLLPSFRSTYRMK
NCU06271.1	IS----RPNTPKVEPIAKAEGTPKAGTSKSSS-------SLKLPPPNLVLPSFKNVYRMK
FG02562.1 	SSPPVSGTSTPTRTATPTQDTDRASAKIAPSDQSIAESETSAKASGNLLLPSFSSTYRMK
AN0093.1  	LS----GPPLPDNVVSKSAGAPEALVPAAPEAS-----KQLQKIIPNQVLPKFRDTFALQ
          	 :     .  .      .            . .     .       * :**.* ..: ::

MG05259.1 	ENPSIIQQIAQFLLRTQQKPQANHAFLTKEPPKIQKALAIGVHGLFPATYLRPMIGQPTG
NCU06271.1	ENPSIVRQIKNYLLWT-QQPAPKHVNIAREPPKIKKAIAFGVHGLIPTYYLRPITGQPTG
FG02562.1 	DNPSIVKQLTQFLLRTQQTP-PNHVFRADKPPNIKKAIAIGVHGLFPATYLRPMIGQPTG
AN0093.1  	DRPTLLQSLGRFLHYS-KEPSNKHVYKLAYPQRIKKALAIGIHGYFPAPLIRSVLGQPTG
          	:.*::::.: .:*  : : *  :*.     * .*:**:*:*:** :*:  :*.: *****

MG05259.1 	TSIRFANHAADSIRRWADSHGCQDCEIEKVALEGEGKIGERVENLWKLLLNWIESIRKAD
NCU06271.1	TSIKFANHGAEAIRRWAESHGCGDCEIDKVALEGEGKIDERVENLWKLLLNWIDKIRQAD
FG02562.1 	TSLRFAALGAEGIRRWAEAHGCGDCKIEKVALEGEGKIQDRVDNLWKLMLNWIDHIRNAD
AN0093.1  	TSVRFSTLAAEAIRKYTDDQGY-TCEIQKIALEGEGRIAERVDLLWKLLLNWMEEIRSAD
          	**::*:  .*:.**:::: :*   *:*:*:******:* :**: ****:***:: **.**

MG05259.1 	LILVACHSQGVPVSIMLVAKLIELGIIT-TPRIGVCAME1--------------------
NCU06271.1	VVIMACHSQGVPVSIMLLAKLYELGVISPTAKVGVCAMA~GVSLGPFPDYKTGLGILMGS
FG02562.1 	FVLIACHSQGVPVSIMLLEKLIDLGIIT-NAKVGVCAMA~GVALGPFPDYKSSL--LMGS
AN0093.1  	FILVACHSQGVPVGIMLVAKLILFGCIN-ANRVGVCAMA~GVNLGPFADYKSR--WISGS
          	.:::*********.***: **  :* *.   ::*****  .   .. .. .:      .:

MG05259.1 	-RPLEQFGNPESDVSKRYEAALKAVLEYGVRVTYIGSIDDQV~VPLE0SALHSPANHPLL
NCU06271.1	AAELWEFGDPQSEVSRKLEAAVKAVLHYGARITYIGSIDDQL~VPLE~SAIYAPAHHPYI
FG02562.1 	AAELWDFGNAESDNSKHFEHALKRVVDYGARVTFIGSIDDQL0S---~-AVYSPANHPYI
AN0093.1  	AGELFEFALPYSKVSKDYESALRTCIDFGVRISYIGSIDDQL~VSLE0SSLFSPVSHPYI
          	:  * :*. . *. *:  * *::  :.:*.*:::*******:  . . :::.:*. ** :

MG05259.1 	FRAVFV2DARVHAADF2IAHLVGFALKLRNMGISDHGLIRELSVPLAGSLYSGAGHSTLY
NCU06271.1	YRAVFI~DGRIHAPDF2IAHLVGFALKLRNLGVTDHGLIRELSVPLAGSLYTGDGHSRLY
FG02562.1 	YRAVFI~DGRVHAPDF~IAHLVGFALKLRNLGVSDHGLIRELSVPLAGSLYSGAGHSRLY
AN0093.1  	YRAAFV~DGRVHAPSF2VSHLVGFALKLRNLGISDHGLIRELSSPLAGSLYTGEGHSRLY
          	:**.*: *.*:**..* ::***********:*::********* *******:* *** **

MG05259.1 	DDPQLYD2LAVSHALETSDVG----------KVPCEIGK-HEG-ITTSNPYHLPWIMRGL
NCU06271.1	DDEQVYD2LAISHALETTDVSPSPTRPNSKSSVPCDMATRQPGTLSNPNPYHLPWIMRGL
FG02562.1 	YDDAVYD~LAIAHALETAPAGPS--------PAPCTISP-RGGSLTSQNPYVLPWIMRGL
AN0093.1  	DEEAVYR2LAIEFALETSPVS----------NAPLHIKR--SYPPPVPNPYILPFAMRGL
          	 :  :*  **: .****: ..           .*  :      . .  *** **: ****

MG05259.1 	LEEDFVKTELSSEVTELLKQFDSWKPVT~KALKDVKYRLEAVRSKL
NCU06271.1	LEEDFVKSELSNEVEDLLKQFDDWKPTT~KALKDVKYRLEAVRSKL
FG02562.1 	LEEDFVRTELSAEADELLKQFDDWKPTN~KALKDVKYRLEAVRSKL
AN0093.1  	LEEEYVRHELYNETMELLKQFDEWKPTK1FGSHLAVFDLLR-----
          	***::*: **  *. :******.***..  . : . : *
```
